# Unusual Presentations of Focal Periphyseal Edema Zones: A Report of Bilateral Symmetric Presentation and Partial Physeal Closure

**DOI:** 10.1155/2015/465018

**Published:** 2015-11-12

**Authors:** Nicholas Beckmann, Susanna Spence

**Affiliations:** Department of Diagnostic and Interventional Imaging, Memorial Hermann, University of Texas Health Science Center, 6431 Fannin Street, No. 2.130B, Houston, TX 77030, USA

## Abstract

Focal periphyseal edema (FOPE) zones are areas of periphyseal edema seen near the time of physeal closure which are believed to be a physiologic phenomenon related to changes in distribution of forces around the physis as it closes. Since the original case series describing these areas of periphyseal edema, there has been little published in regard to FOPE zone outside of review articles. We present a set of three patients identified with focal periphyseal edema zones around the knee and compare our findings with the initial case series. We include a patient presenting with bilateral, nearly symmetric, focal periphyseal edema zones of the proximal tibia physis and a patient with partial closure of the physis at time of presentation, which were not reported in the original case series.

## 1. Introduction

In 2011, Zbojniewicz and Laor described areas of bone marrow edema centered on physes of the knee nearing closure in adolescent patients [1]. They coined these areas of marrow edema focal periphyseal edema, or FOPE, zones. Since their original case series in 2011, FOPE zones have been referred to in a few other review publications [2–4], but, to the best of our knowledge, there have not been any additional published case studies describing the imaging and clinical features of FOPE zones. The three adolescent patients presented in this series have areas of periphyseal edema around the knee consistent with FOPE zones previously described by Zbojniewicz and Laor. However, two of the patients had an imaging appearance of FOPE zones not previously described in their paper. One of the patients had bilateral nearly symmetric FOPE zones while another patient had partial closure of her physis associated with the FOPE zone.

## 2. Materials & Methods

### 2.1. Case 1

**Figure 1:**
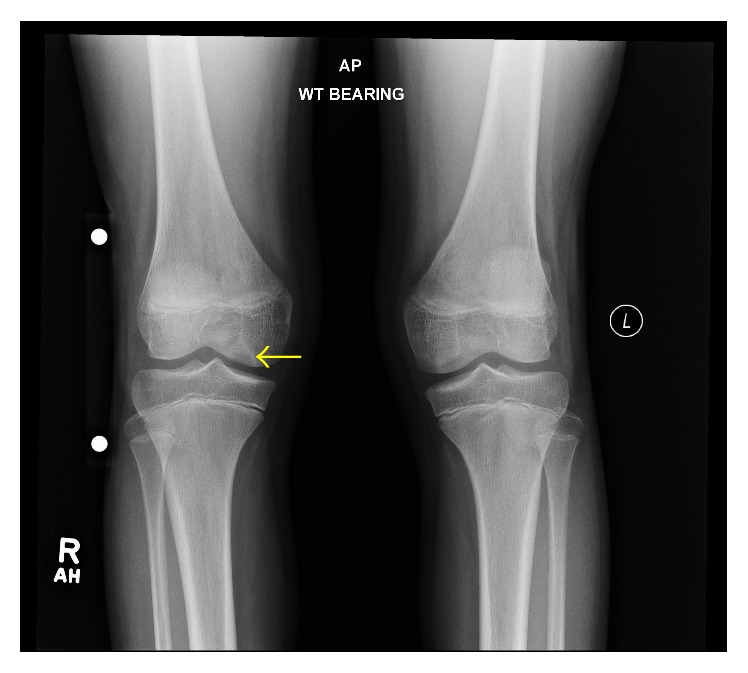
A 13-year-3-month-old female with FOPE zones at both proximal tibiae. Findings: AP radiograph of the bilateral knees at time of initial presentation shows a subtle lytic lesion abutting the articular surface of the right medial femoral condyle (arrow). The radiographs were initially interpreted as normal. Technique: AP radiograph kVp = 77, mAs = 4.

An adolescent female softball player presented with gradually worsening right anterior knee pain and knee stiffness of several months duration. She had focal tenderness in the right infrapatellar region on palpation and mild pain during knee flexion. Initial radiographs of the right knee (Figure 1) were interpreted as normal. The patient was treated with a presumed diagnosis of patellar tendinitis and instructed to undergo a brief period of rest followed by physical therapy with gradual return to normal activity.

**Figure 2:**
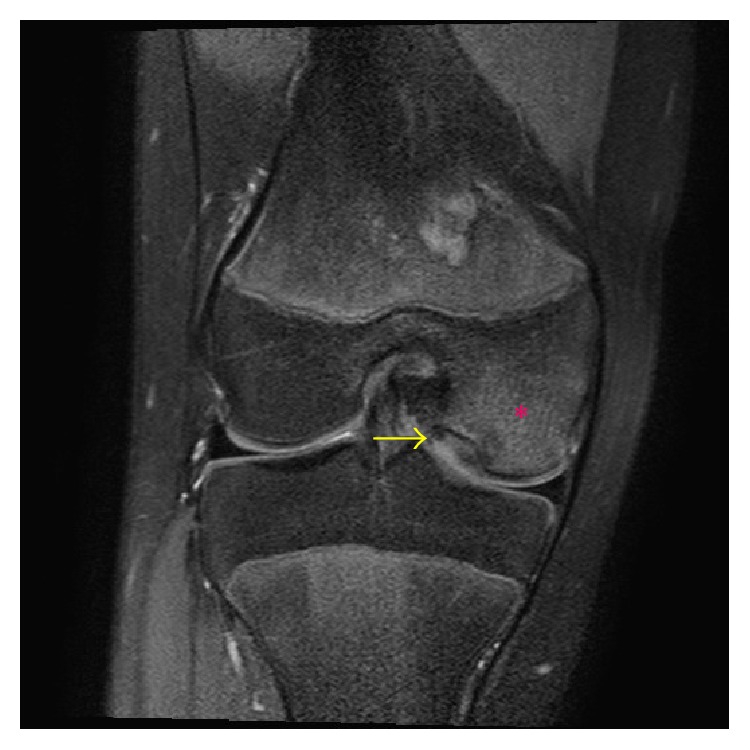
A 13-year-3-month-old female with FOPE zones at both proximal tibiae. Findings: coronal proton density fat-saturated image from right knee MRI obtained 9 months after initial presentation shows an osteochondral lesion (arrow) with mild surrounding marrow edema (asterisk) of the medial femoral condyle corresponding to subtle lytic lesion present on initial radiographs. Technique: coronal PD fat-saturated MRI (TR = 2,825.7, TE = 30), ST = 2.5 mm, spacing = 3.264 mm, FOV = 14 cm, and matrix = 312 × 244.

She returned 9 months later with persistent anterior right knee pain and mild knee pain with deep knee flexion. At this time, mild medial joint line tenderness was also elicited on palpation. Due to the medial joint line tenderness, a right knee MRI was performed to assess for medial meniscal pathology. A 1.6 × 1.4 cm osteochondral lesion of the medial femoral condyle was identified on MRI with mild marrow edema and cystic change at the interface between the osteochondral lesion and femoral condyle (Figure 2). There was no defect in the overlying cartilage or fluid-like signal at the interface of the osteochondral lesion and femoral condyle. The remainder of the MRI exam was normal.

**Figure 3:**
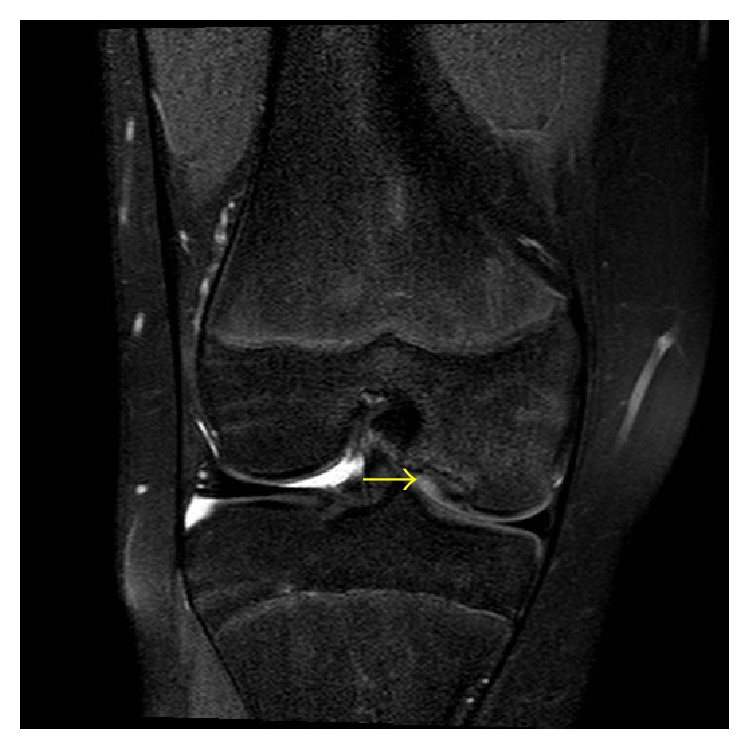
A 13-year-3-month-old female with FOPE zones at both proximal tibiae. Findings: coronal proton density fat-saturated image from follow-up of right knee MRI obtained 6 months after initial MRI shows decrease in the marrow edema surrounding the osteochondral lesion of the medial femoral condyle. The osteochondral lesion (arrow) is otherwise unchanged in appearance. Technique: coronal PD fat-saturated MRI (TR = 2,472.4, TE = 30), ST = 2.5 mm, spacing = 3.3 mm, FOV = 14 cm, and matrix = 312 × 244.

**Figure 4:**
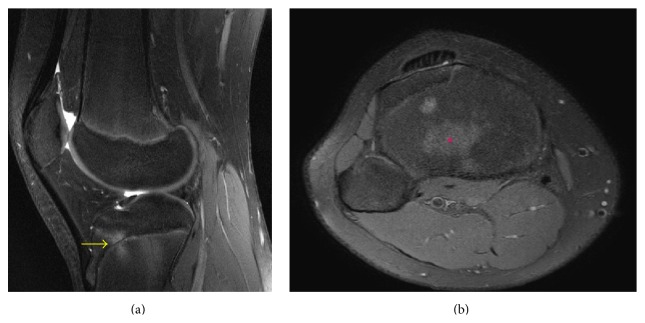
A 13-year-3-month-old female with FOPE zones at both proximal tibiae. Findings: (a) sagittal proton density fat-saturated image on the 6-month follow-up MRI of the right knee shows interval development of 9 mm in diameter area of periphyseal edema on both the epiphyseal and metaphyseal sides of the proximal tibia physis (arrow). The proximal tibia physis is narrowed but remains open. (b) Axial proton density fat-saturated image at the level of the proximal tibia physis shows the periphyseal edema located at the anterolateral aspect of the central physis (asterisk). Technique: (a) sagittal PD fat-saturated MRI (TR = 2,958.3, TE = 30), ST = 3 mm, spacing = 3.3 mm, FOV = 14 cm, and matrix = 364 × 261; (b) axial PD fat-saturated MRI (TR = 2,843.3, TE = 30), ST = 3 mm, spacing = 3.3 mm, FOV = 15 cm, and matrix = 376 × 297.

**Figure 5:**
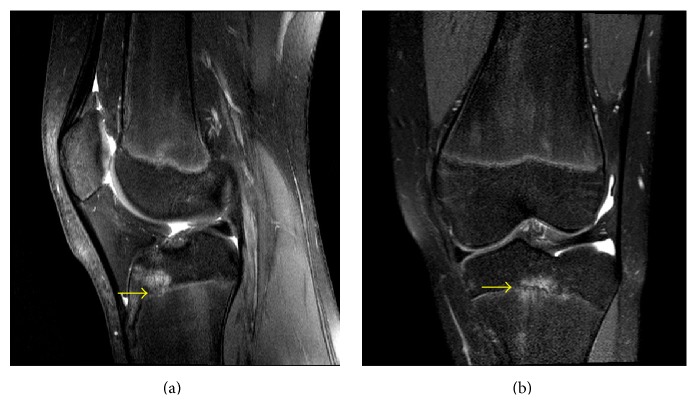
A 13-year-3-month-old female with FOPE zones at both proximal tibiae. Findings: (a) sagittal proton density fat-saturated image of the left knee shows 13 mm in diameter area of periphyseal edema at the metaphyseal side of the proximal tibia physis (arrow). The proximal tibia physis is narrowed but remains open. (b) Coronal proton density fat-saturated image shows the periphyseal edema to be on both sides of the proximal tibia physis. The coronal image better demonstrates the periphyseal edema involvement of both the epiphyseal and metaphyseal sides of the physis (arrow). Technique: (a) sagittal PD fat-saturated MRI (TR = 2,959.1, TE = 30), ST = 3 mm, spacing = 3.3 mm, FOV = 14 cm, and matrix = 364 × 261; (b) coronal PD fat-saturated MRI (TR = 2,504.2, TE = 30), ST = 2.5 mm, spacing = 3.3 mm, FOV = 15 cm, and matrix = 312 × 244.

**Figure 6:**
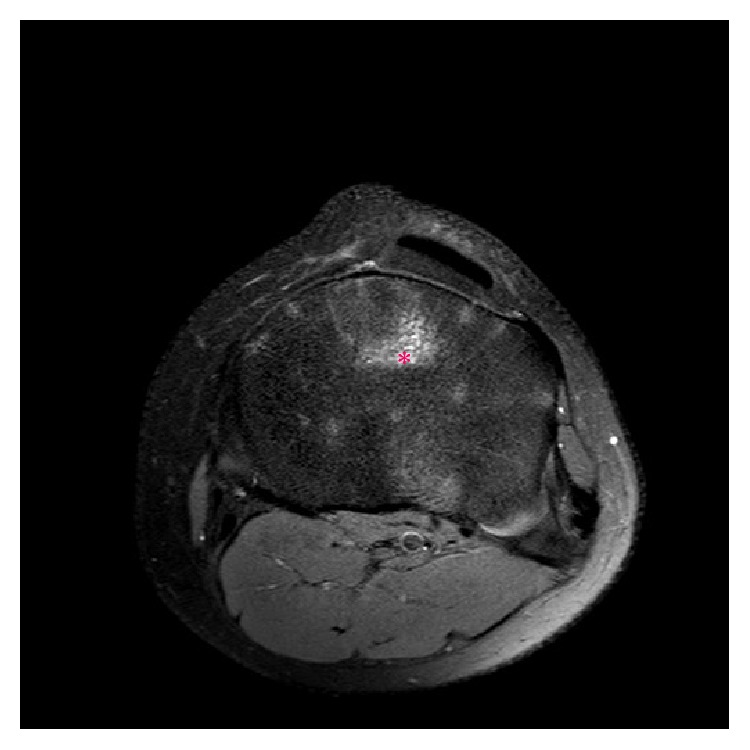
A 13-year-3-month-old female with FOPE zones at both proximal tibiae. Findings: axial proton density fat-saturated image at the level of the proximal tibia physis shows the periphyseal edema located at the anterocentral aspect of the central physis (asterisks). Technique: axial PD fat-saturated MRI (TR = 2,843.2, TE = 30), ST = 3 mm, spacing = 3.3 mm, FOV = 15 cm, and matrix = 376 × 297.

The patient's medial joint line pain improved, but she continued to have mild anterior knee pain. A repeated MRI of the right knee was performed 6 months after the initial MRI to assess for changes in the osteochondral lesion. MRI of the left knee was performed at the same time due to the patient complaining of vague anterior left knee pain similar to the presenting right knee pain, which raised clinical concern for an occult osteochondral lesion of the left knee. She was at chronologic age of 13 years and 3 months at the time of the bilateral knee MRI. The osteochondral lesion of the right medial femoral condyle was unchanged in appearance between the two MRI exams (Figure 3). However, in the interim, a 9 mm focal periphyseal edema (FOPE) zone developed in the anterolateral aspect of the central proximal right tibia (Figure 4). A similar appearing 13 mm FOPE zone was present in the anterior aspect of the central proximal left tibia on the left knee MRI (Figures 5 and 6). While the distribution of FOPE zone periphyseal edema was equal on the epiphyseal and metaphyseal sides of the proximal right tibia, the periphyseal edema was more exuberant on the epiphyseal side of the proximal left tibia. The remainder of the left knee MRI was normal. The proximal tibial physis was narrowed, but open, bilaterally.

It was uncertain whether the patient's bilateral anterior knee pain was due to the FOPE zones or another etiology not elucidated on the MRI. However, the patient pain resolved after a several-week period of rest. She was released to continue activity as tolerated with instruction to return if pain increased. She did not return for follow-up.

### 2.2. Case 2

An adolescent female dancer and cheerleader at chronological age of 14 years and 2 months presented with right knee pain beginning after twisting her knee one month prior to presentation. The knee pain was initially medial but had migrated to the anterior knee by the time of presentation. The patient was focally tender to palpation in the infrapatellar region on physical exam and had patella crepitus. She had diminished flexion due to pain.

**Figure 7:**
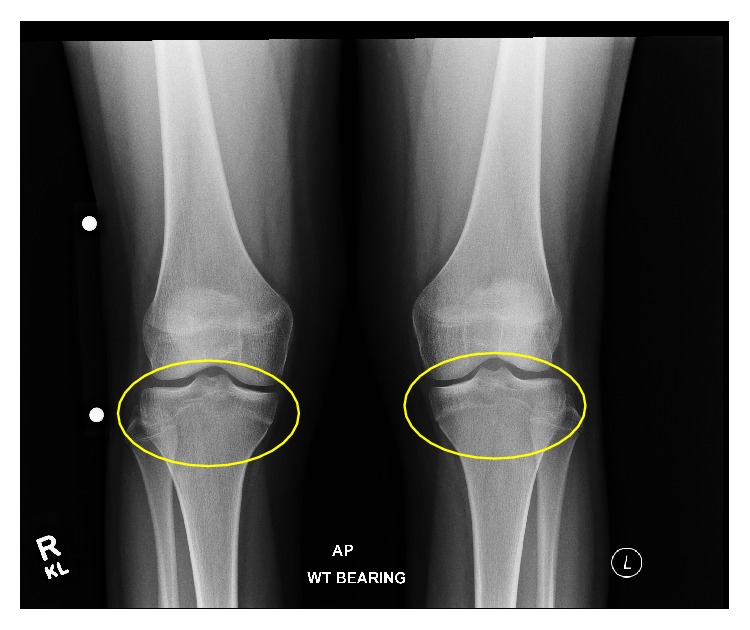
A 14-year-2-month-old female with FOPE zone of the proximal tibia. Findings: AP radiograph of both knees shows bilateral symmetric partial closure of the central portion of the proximal tibial physis (ovals). Technique: AP radiograph kVp = 70, mAs = 7.

**Figure 8:**
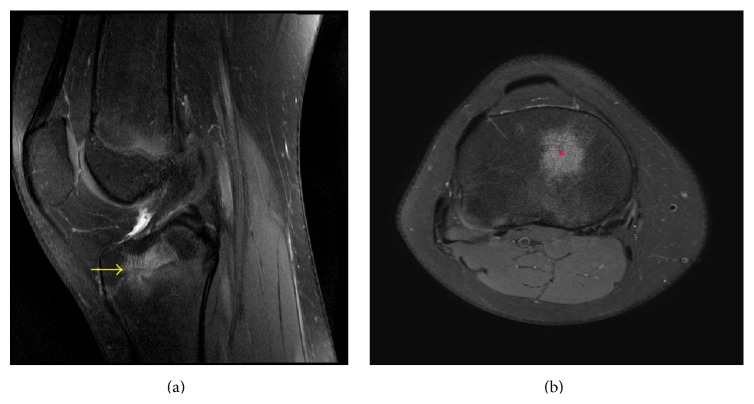
A 14-year-2-month-old female with FOPE zone of the proximal tibia. Findings: (a) sagittal proton density image shows 14 mm in diameter area of periphyseal edema involving both sides of the proximal tibia physis (arrow). Edema is greater along the epiphyseal side of the physis. (b) Axial proton density image at the level of the proximal tibia physis shows the area of periphyseal edema within the anteromedial aspect of the central proximal tibia (asterisk). Technique: (a) sagittal PD fat-saturated MRI (TR = 2,922.8, TE = 30), ST = 3 mm, spacing = 3.3 mm, FOV = 14 cm, and matrix = 364 × 261; (b) axial PD fat-saturated MRI (TR = 2,803.1, TE = 30), ST = 3 mm, spacing = 3.3 mm, FOV = 15 cm, matrix = 376 × 297.

**Figure 9:**
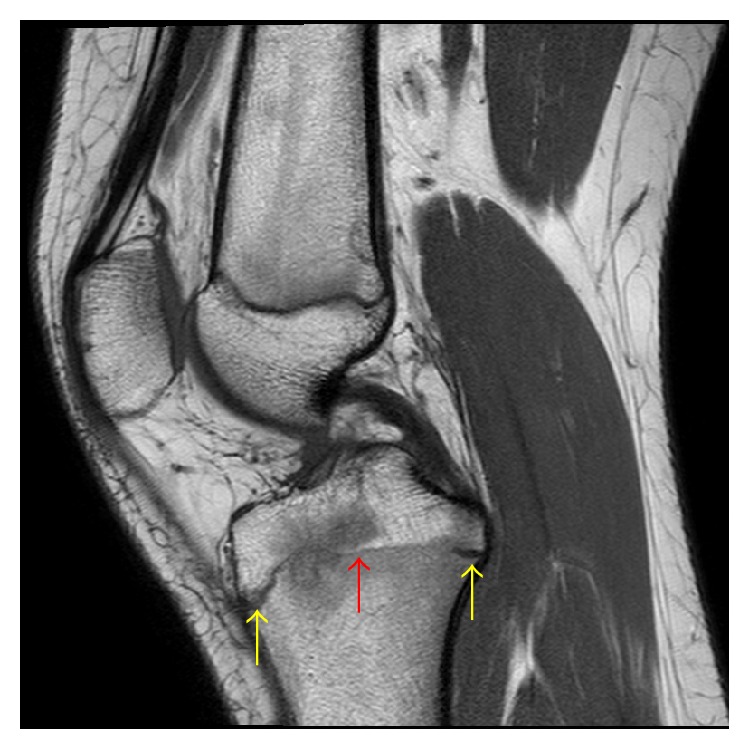
A 14-year-2-month-old female with FOPE zone of the proximal tibia. Findings: Sagittal T1 weighted image shows partial closure of the proximal tibia physis centrally (red arrow) with the physis still open along the periphery of the tibia (yellow arrows). The distal femur physis is narrowed but still open by comparison. Low T1 signal intensity corresponding to the periphyseal edema is again seen spanning the proximal tibia physis. Technique: sagittal T1 MRI (TR = 621.991, TE = 15), ST = 3 mm, spacing = 3.3 mm, FOV = 14 cm, and matrix = 340 × 277.

There was no abnormality on presenting knee radiographs, which showed bilateral symmetric partial closure of the central portion of the proximal tibial physis (Figure 7). Right knee MRI was performed to assess for possible patellofemoral chondromalacia. A 14 mm FOPE zone was present in the anteromedial aspect of the central proximal tibia physis on MRI (Figure 8). The right knee MRI was otherwise unremarkable. The proximal tibia physis was partially closed centrally on the knee MRI (Figure 9).

The patient underwent a short period of rest followed by physical therapy with gradual return to activity. She reported significant improvement in knee pain 2 months after initial presentation and has not returned for follow-up.

### 2.3. Case 3

An adolescent female at chronological age of 13 years and 10 months presented with acute right knee pain after hyperextending her knee while playing basketball. She felt a “pop” during the hyperextension and had been unable to bear weight after injury. On physical examination, she had lateral joint line pain without ligamentous laxity. There was limited knee range of motion, and a moderate joint effusion was present.

**Figure 10:**
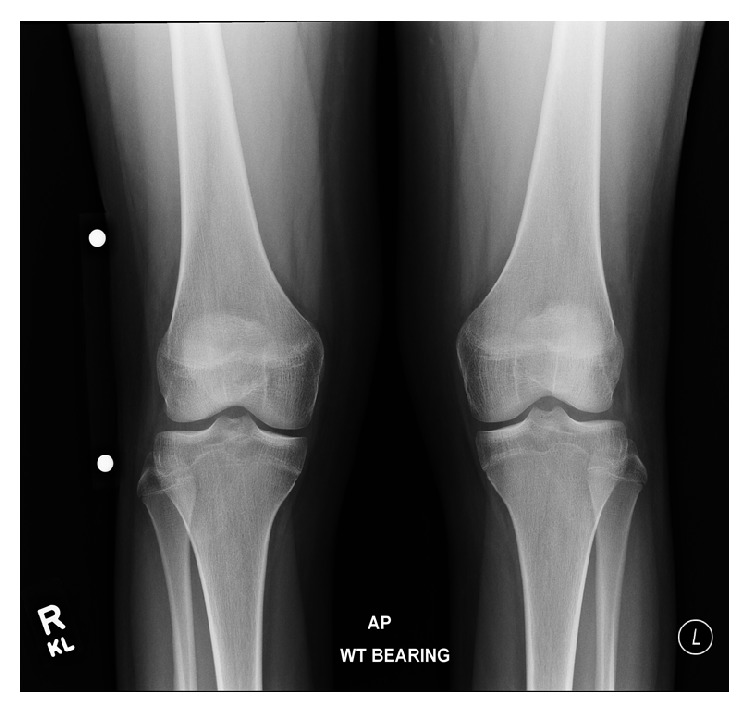
A 13-year-10-month-old female with FOPE zone of the distal right femur. Findings: presenting radiographs of the knees were normal. There is partial closure centrally of the bilateral proximal tibia physes. The bilateral distal femur physes are narrowed but remain open. Technique: AP radiograph kVp = 70, mAs = 8.

**Figure 11:**
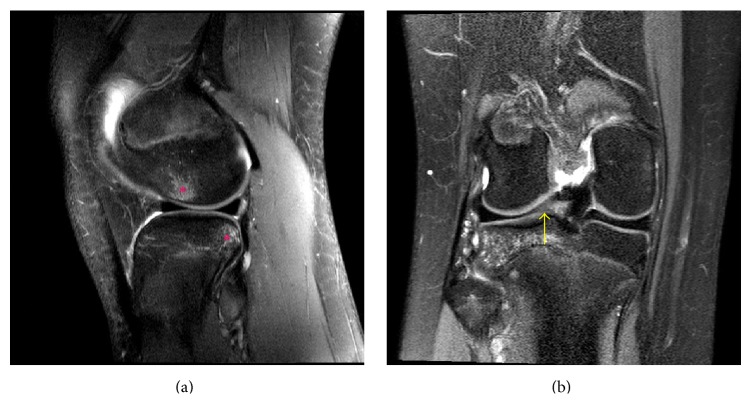
A 13-year-10-month-old female with FOPE zone of the distal right femur. Findings: (a) sagittal proton density fat-saturated image shows pivot-shift pattern of bone marrow edema at the weight-bearing aspect of the lateral femoral condyle and posterior aspect of the lateral tibial plateau (asterisks). (b) Coronal proton density fat-saturated image shows increased signal in the lateral meniscus posterior horn root ligament (arrow) consistent with tearing of the ligament. Tear of the root ligament was confirmed on arthroscopy. Technique: (a) sagittal PD fat-saturated MRI (TR = 2,557, TE = 30), ST = 3 mm, spacing = 3.3 mm, FOV = 14 cm, and matrix = 364 × 261; (b) coronal PD fat-saturated MRI (TR = 2,826.1, TE = 30), ST = 2.5 mm, spacing = 3.3 mm, FOV = 15 cm, and matrix = 312 × 244.

**Figure 12:**
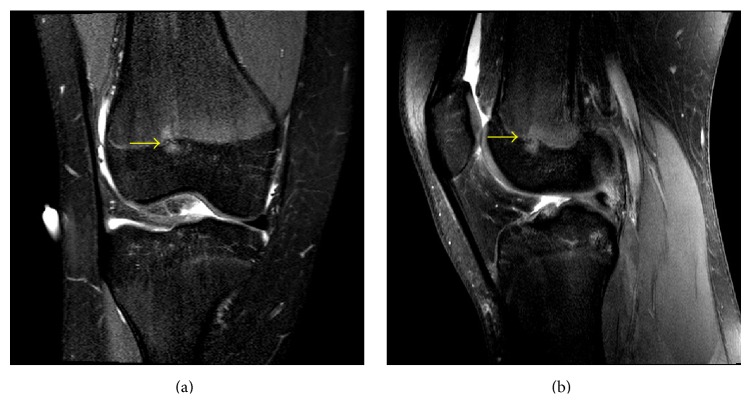
A 13-year-10-month-old female with FOPE zone of the distal right femur. Findings: (a) coronal proton density fat-saturated image shows a 5 mm area of periphyseal edema slightly eccentrically located on both sides of the distal femur physis (arrow). The edema is slightly more prominent on the epiphyseal side of the physis. (b) Sagittal proton density fat-saturated image again shows the eccentrically located area of periphyseal edema in the distal femur physis (arrow) with the edema again slightly more prominent on the epiphyseal side. The distal femur physis is narrowed but remains open. Technique: (a) coronal PD fat-saturated MRI (TR = 2,826.1, TE = 30), ST = 2.5 mm, spacing = 3.3 mm, FOV = 15 cm, and matrix = 312 × 244; (b) coronal PD fat-saturated MRI (TR = 2,826.1, TE = 30), ST = 2.5 mm, spacing = 3.3 mm, FOV = 15 cm, and matrix = 312 × 244.

Presenting radiographs of the right knee were normal (Figure 10). MRI of the right knee showed pivot-shift bone marrow contusion pattern in the lateral compartment and root ligament avulsion of the lateral meniscus posterior horn (Figure 11). A 5 mm FOPE zone was also present in the central portion of the distal femur physis (Figure 12). The distal femur physis was still open.

She underwent 2 months of conservative management without relief in symptoms. The patient subsequently had knee arthroscopy for repair of the lateral meniscus posterior horn root ligament. The right knee pain and joint effusion resolved after surgery with return of normal range of motion by 3 months after meniscal repair. The patient has not returned for additional follow-up.

## 3. Discussion

The etiology of FOPE zones is uncertain; however, it is postulated that these areas of edema may be physiologic and related to normal physeal closure [1]. Physeal closure starts centrally and proceeds peripherally. The central portion of the physis has increased susceptibility to trauma, which is believed to be due to decreased elasticity of the central physis relative to the periphery during the early closure of the central physis [2]. This decreased elasticity could also potentially predispose the central physis to increased stress during physeal closure. Bone marrow edema on MRI has been shown to be a manifestation of increased stress placed on the bone [5], which could explain the appearance of marrow edema around the narrowed central physis typically seen in FOPE zones. While some FOPE zones may be physiologic, two of the three patients we reported had knee pain in the region of the FOPE zones without other explanations for pain on their knee MRIs, suggesting that the FOPE zones may represent a painful phenomenon related to mechanical strain placed on the physis during physeal closure.

In Zbojniewicz and Laor's paper, all 12 patients with FOPE zones were between 12 and 16 years of age. This age range coincides with the expected age of physeal closure at the knee, which supports the theory that FOPE zones are related to physeal closure. The physes of the knee typically close between 12 and 15 years of age with 5% of patients having closed physes by 12 years of age and 94% of patients having closed physes at 15 years of age [6]. All three of our patients were between the ages of 13 and 15 at time of presentation.

No gender or racial predilection has been described for FOPE zones. However, seven of the 12 patients in the case series by Zbojniewicz and Laor were female, and all three of the patients in our series were female. This suggests that there could be a higher incidence of FOPE zones in females.

It is uncertain whether FOPE zones can present with clinical symptoms. Five of the twelve patients in Zbojniewicz and Laor's study had no MRI finding beyond the FOPE zone to explain their pain, suggesting that FOPE zones may be a source of pain. However, a little more than half of the patients in the study did have other explanations for their pain, and bone marrow edema has been shown to be an incidental finding in asymptomatic, active adolescents [7]. In our series, patient #3 had a clear source for her pain other than the FOPE zone, patient #2 had no clear source of her pain, and patient #1 had a potential source of knee pain on the right and no clear source of pain on the left one. It should be noted that the anterior right knee pain in patient #1 was not clearly referable to the right medial femoral condyle osteochondral lesion.

FOPE zones appear as areas of T1 hypointense and T2 hyperintense marrow edema on both the epiphyseal and metaphyseal sides of the physis with the areas of edema demonstrating contrast enhancement [2]. The edema is typically slightly eccentrically centered in the central portion of the physis with a great deal of variability in the extent of edema present. The physis has previously been described as being narrowed but open in the region of periphyseal edema [1, 2]. However, in one of our cases, the central portion of the physis was closed with surrounding periphyseal edema, which has not been previously described. Multiple physes can be involved at the same time [1, 2].

A FOPE zone is a self-limited process that requires no imaging follow-up or diagnostic intervention. FOPE zones likely resolve quickly once physeal closure occurs, although our patient with partial closure of her physis demonstrates that FOPE zones can persist during physeal closure. While there is no standard treatment for FOPE zones, symptomatic FOPE zones can generally be treated with periods of rest and activity cessation similar to the treatment for stress reaction of the bone. A clinical summary of FOPE zones is included at the end of the paper (Table 1).

The physis acts as a relative barrier, preventing the spread of most tumors from the metaphysis to the epiphysis [8], although aggressive pediatric tumors such as Ewing's or osteosarcoma can cross the physis [9] and could be considered in the differential for FOPE zones. However, the typical presenting age and location, symmetric uniform appearance of marrow edema on both sides of the physis, and lack of aggressive features should allow for the distinction between FOPE zones and malignancy without much difficulty. Malignancy will often demonstrate heterogenous enhancement while the enhancement of edema in FOPE zones tends to be relatively uniform.

Infection is more likely to present with enhancing periphyseal edema that could mimic a FOPE zone. FOPE zones will lack the exuberant marrow edema and intraosseous fluid collections frequently seen with infection, and the clinical presentation as well as elevated inflammatory markers typically makes differentiating between infection and FOPE zone possible. A table summarizing the MRI characteristics of FOPE zones and the major differential considerations is included (Table 2).

Chronic physeal stress injury could present with chronic knee pain and physeal signal changes similar to a FOPE zone. On MRI, though, FOPE zones demonstrate periphyseal edema around a narrowed physis, while chronic physeal stress injuries appear as physeal widening, typically without periphyseal edema [10].

## 4. Conclusion

FOPE zones are a phenomenon seen at time of physeal closure, typically in the knee. While some FOPE zones may be physiologic, our cases would suggest that FOPE zones may be a possible source of pain potentially from alterations in mechanical stress across the physis as it closes. FOPE zones typically are seen as a unilateral process prior to physeal closure; as our cases show, FOPE zones can occur both after the physis begins to close and as a bilateral symmetric process. FOPE zones should be recognized as a benign self-limited entity and not mistaken for an aggressive process crossing the physis.

## Figures and Tables

**Table 1:** FOPE zone clinical summary.

Etiology	Unknown. Likely due to mechanical stress placed on the physis during early physeal closure.

Incidence	Rare. Only a small case series reported in the literature.

Gender ratio	Unknown. There may be a higher incidence in females.

Age of predilection	12–16 years old.

Risk factors	No known risk factors.

Treatment	No treatment for asymptomatic lesions. Symptomatic lesions treated conservatively with protected weight bearing.

Prognosis	Excellent. Self-limited process that likely resolves shortly after physeal closure.

Imaging findings	Focal enhancing bone marrow edema on both sides of a central portion of a narrowed or partially closed physis.

**Table 2:** Differential diagnosis MRI characteristics.

Pathology	MRI, T1	MRI, T2	MRI enhancement
FOPE zone	Relatively symmetric focal decreases signal intensity on both sides of central portion of narrowed or partially closed physis.	Relatively symmetric focal increased signal intensity on both sides of central portion of narrowed or partially closed physis.	Avid, uniform enhancement of areas of bone marrow edema.

Infiltrative bone tumor	Low signal typically on one side of the physis. Low signal distribution will be asymmetric if tumor does cross physis.	High signal typically on one side of the physis. High signal distribution will be asymmetric if tumor does cross physis.	Typically avid enhancement of areas of increased T2 signal. Enhancement is often heterogeneous.

Osteomyelitis	Low signal that may cross the physis. Distribution of low signal across physis is typically asymmetric. Other findings of infection such as cortical disruption, fluid collections, and periosteal reaction are often present.	High signal that may cross the physis. Distribution of high signal across physis is typically asymmetric. Other findings of infection such as cortical disruption, fluid collections, and periosteal reaction are often present.	Typically avid, heterogenous enhancement. Nonenhancing fluid collections may be present.

Chronic physeal stress injury	Widening of the physis typically without periphyseal low signal.	Widening of the physis typically without periphyseal high signal.	Typically no increased periphyseal enhancement.

## Abbreviations

cm: Centimeter
FOPE: Focal periphyseal edema
FOV: Field of view
kVp: Peak kilovoltage
mAs: Milliampere-second
mm: Millimeter
MRI: Magnetic resonance imaging
PD: Proton density
ST: Slice thickness
TR: Relaxation time
TE: Echo time.
